# Experiences of support in working toward personal recovery goals: a collaborative, qualitative study

**DOI:** 10.1186/s12888-016-1133-x

**Published:** 2016-11-25

**Authors:** Eva Biringer, Larry Davidson, Bengt Sundfør, Torleif Ruud, Marit Borg

**Affiliations:** 1 Helse Fonna Local Health Authority, P.O. Box 2170, N-5504 Haugesund, Norway; 2 Regional Research Network on Mood Disorders (MoodNet), Bergen, Norway; 3 Department of Psychiatry, Yale University School of Medicine, New Haven, CT USA; 4 Division of Mental Health Services, Akershus University Hospital, Lørenskog, Norway; 5 Institute of Clinical Medicine, University of Oslo, Oslo, Norway; 6 Faculty of Health Sciences, University College of Southeast Norway, Drammen, Norway

**Keywords:** Mental health, Recovery, Everyday life, Hope, Expectancy, Personal goal, Service user experience, Patient satisfaction, Patient-centred care, Psychotherapy, Mental health services

## Abstract

**Background:**

Recovery can be understood as a subjective process guided by personal expectations, goals and hopes. The aim of the study was to explore how persons using a Community Mental Health Centre (CMHC) experienced that their expectations for treatment, and goals and hopes for recovery were supported by the health professionals during treatment.

**Methods:**

Employing a hermeneutic–phenomenological approach, eight service users were interviewed about their expectations for treatment and their goals and hopes for recovery at the start of their contact with health professionals at a CMHC. Two years later, they were re-interviewed about their experiences of treatment and support from the health professionals in their work towards these goals and hopes. A collaborative approach was adopted. A co-researcher with lived experience took part in all stages of the study. Data were analysed by means of a data-driven stepwise approach in line with thematic analysis.

**Results:**

Five themes reflecting how participants experienced support from health professionals at the CMHC in their work towards their recovery goals were elicited, as follows: developing an understanding of oneself and one’s mental health problems; learning how to change feelings and behaviours; being ‘pushed’ into social arenas; finding helpful medication; and counselling in family, practical and financial issues. The participants’ expectations about counselling with regard to longer-term family, practical, and financial challenges were insufficiently met by the CMHC. In the experience of the service users, recovery occurred within the context of their everyday life with or without the support of their professional helpers.

**Conclusions:**

To facilitate recovery, health professionals should acknowledge the service user’s personal goals and hopes and take a more comprehensive and longer-term approach to his or her needs and desires. Acknowledging and facilitating recovery goals by offering counselling with regard to family, practical and financial issues seems particularly important.

**Electronic supplementary material:**

The online version of this article (doi:10.1186/s12888-016-1133-x) contains supplementary material, which is available to authorized users.

## Background

Recovery is conceptualised as both a process and an outcome, and improvement is not only reflected in changes in the state of the disorder (resolution) but can just as much be seen as an adjustment of life to work around the disorder (readjustment) or an adaptation to living with the disorder (redefinition) [1]. Hope and optimism about the future, motivation to change, and empowerment are core features of personal recovery [2–5]. Thus, healthcare professionals and researchers who take a recovery-oriented position acknowledge and support the person in his or her work to achieve personal aims, desires, hopes, and dreams in life [6–8]. Taking such a supportive attitude involves focusing on the individual’s right to make personal decisions from an array of options about all aspects of his or her own recovery process, including areas like desired goals and outcomes and preferred services used to achieve the desired outcomes [9]. International best practice in supporting recovery identifies four domains of action, as follows: developing a working relationship with the individual, providing treatments to support personally-defined recovery, maintaining an organisational commitment to recovery, and promoting citizenship among persons in recovery [10]. There is a need for research into how and to what extent persons suffering from mental health problems experience the care they receive as helpful in their work to achieve their personal goals. These issues are important from both psychotherapeutic and rehabilitation perspectives. More knowledge in the field would help to improve treatment and further inform health service development.

**Fig. 1:**
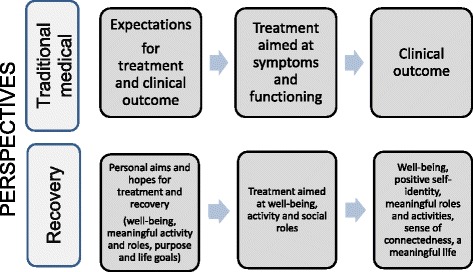
Traditional medical perspective versus recovery perspective concerning expectations, treatment, and outcomes in mental health care

Ideally, the process of recovery should be explored within longitudinal designs focusing on the person’s self-defined outcomes, personal change, and contextual factors. However, longitudinal studies evaluating how mental health services respect and promote service users’ personal expectations for treatment and aims for recovery are hard to find. In a cross-sectional study by Lakeman et al. [11], ‘experts-by-experience’ rated listening to and respecting the person’s viewpoints; conveying a belief that recovery is possible; and recognising, respecting, and promoting the person’s resources and capacity for recovery as the most valued competencies that mental health workers can have in supporting recovery. In one study conducted in an outpatient setting, service users expressed that health professionals tended to focus more on symptoms and failed to engage with their ‘real’ problems [12]. Earlier research exploring service users’ experiences has shown a discrepancy between the perspective of the service user and the more traditional ‘medical’ perspective in terms of treatment goals [13–15]. For many service users, the goals of promoting well-being and energy, social relations, meaningful activity (such as a job), and participation in society may be just as important as the amelioration of disturbing symptoms (Fig. 1) [3, 13–20].

**Fig. 2:**
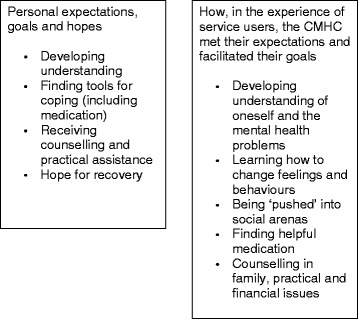
Themes representing participants’ expectations, goals and hopes at start of treatment (Biringer et al. [22]) and how they perceived that the health professionals at the Community Mental Health Centre met and supported these during treatment

The present longitudinal study explores lived experiences of individuals using a Community Mental Health Centre (CMHC) as one way to obtain assistance for their mental health issues. In Norway, CMHCs are the most common type of organisational unit within public specialist mental health care, but similar types of community-based services offering specialist care exist in many Western countries. The key elements that these mental health services are supposed to promote are a focus on service users’ needs, user involvement, coping and empowerment, comprehensiveness, active participation in society, collaboration between stakeholders, and planning and continuity [21]. At the beginning of their contact with the CMHC, participants in the present study hoped to receive help to develop their understanding of their mental health problems, find tools for coping, and access counselling and practical assistance with issues that needed to be solved to create a stable base for their recovery (Fig. 2) [22]. Their expectations for what would happen during treatment, personal goals for recovery, and life goals were tightly interwoven, and recovery was described as a contextual process taking place within the participants’ daily lives and surroundings. The aim of the present study was to provide a deeper understanding of how service users’ expectations for treatment and goals and hopes for recovery at the beginning of their contact with the CMHC was supported by the health professionals during their treatment at the CMHC. To this end, participants were re-interviewed about their experiences at a CMHC two years after they started treatment there.

## Methods

### Setting

The study took place at a typical Norwegian CMHC with an outpatient clinic, outreach services, and two inpatient units for adults. A majority of persons in the region needing assessment or treatment at specialist care level are referred to the CMHC by their general practitioners. Treatment at the outpatient clinic involves regular meetings with a health professional (psychologist, psychiatrist, mental health nurse, occupational therapist, or social worker) in his or her offices. During these meetings, the health professional typically offers more or less specific psychotherapeutic approaches, medications, and counselling. The centre also runs psycho-educational groups that focus on mastery of stress, emotions, and relational problems through the use of cognitive techniques, mindfulness, and relaxation techniques.

### Design

This study is longitudinal, with interviews at two points in time. Eight participants were interviewed at the beginning of treatment [22, 23] and again 27–30 months later. As personal recovery typically is understood as a long-lasting process, a long follow-up interval was chosen in order to facilitate exploration of the person’s recovery process over time.

### Sample

Aiming at including 10 service users, participants were recruited by their therapist at the CMHC at the start of treatment. The first 10 persons who provided informed consent to participate and who were available were included in the study. These participants were interviewed at the start of treatment. Out of these 10, eight agreed to participate again two years later. These eight participants were 18–54 years of age (mean 32 years); four were women and four men. Three were inpatients, while five were outpatients. Several reported anxiety and depressive symptoms, one chronic bodily pain, two psychotic symptoms, one alcohol addiction and anxiety, and one addiction to a combination of alcohol and other substances. After the first interview, the outpatients continued to have contact with their health professional at the CMHC, two participants attended psycho-educational groups, and one participant received regular home visits by the outreach team of the CMHC. At the time of follow-up, six participants had completed their contact with the centre in agreement with their health professionals, and two participants were still using the services of the CMHC.

### Data collection and analysis

As each recovery journey is personal and unique, and as we aimed at exploring subjective experiences in people’s everyday lives, we chose a hermeneutic–phenomenological approach [24–26]. A central element in phenomenology is that lived experience provides meaning to every individual’s interpretation and understanding of a phenomenon [25–27]. Therefore, in order to facilitate the exploration of the idiosyncratic experiences and understandings of each participant, and his or her everyday life contexts, the semi- structured interview guide for the in-depth interviews included only themes and questions that were open-ended in nature. The interview guide was developed in collaboration with service users. The interview guide was broad and aimed at exploring most areas of everyday life, with emphasis on social as well as practical issues, and also health and illness experiences. The baseline interview included questions about experiences of mental health problems, expectations of treatment at the CMHC, and personal goals for treatment and life in general. Two years later, the interview guide focused on the participants’ experience of being supported by health services since the first interview, how helpful the CMHC -treatment and support had been in their work to achieve the personal goals identified at the first interview, and experiences of support from other people and places. Further, it included questions about current life activities, events, and contextual factors. Examples of questions focusing on experience of helpful help from the health professionals are: ‘What, in your experience, have the professional helpers you have encountered [since the last interview] done that was helpful? … Or not helpful? … In what way?’ and ‘The last time we talked you said that your goals for your treatment and life were …..[repetition of the individual participant’s personal goals from two years back in time]. Have you reached any of these goals? … Can you please tell me how? ’

The study had a reflexive–collaborative framework. The aim of service user involvement was to enrich and develop the understanding of the participants’ experiences in a situation where the authors with traditional clinical backgrounds could have their pre-understandings challenged in an ongoing dialogue. A co-researcher (BS) took an active part in interviews, data analysis, and dissemination of results. In the interviews, both participant and co-researcher could interactively explore and give meaning to their personal experiences concerning the issues in question. Interviews were audiotaped and transcribed verbatim. Further information about the methodological approach used in the study can be found in Biringer et al. [23].

Recurring themes analysis was performed across the sample at each time point and longitudinally, with the individual participant as unit of analysis [28–30]. The initial steps of the analysis process were carried out using a data-driven stepwise approach in line with thematic analysis [31]. Searching for meanings and patterns to identify preliminary themes, the primary author read all transcripts at both time points. Using NVivo 9.0, she then systematically coded all text material. Further, to look for recurrence of themes and changes over time from an intra-individual perspective, a table showing evidence for the themes found was produced for transcripts from interviews at both time points with each participant.

In line with a hermeneutic-phenomenological approach, the researchers proceeded through data analysis with the objective of staying close and reflexive to the experiential horizon of the participants [24]. The contents of the preliminary themes were presented to MB and BS in three two-day workshops. During group discussions in which reflexivity was emphasised, a common understanding about semantic and latent constructs underlying the text material and categorisation of the material was reached. When writing the results, the first author used notes from each of the researchers participating in the workshops. To ensure internal validity of findings, two authors (BS and EB) independently compared the results with the original transcripts.

### Ethics, consent and permissions

All participants provided written informed consent. Approval for the study by the Regional Committee for Medical Research Ethics was applied for (ref. no. 2009/1295). The Regional Committee for Medical Research Ethics referred the study to the Norwegian Social Science Data Services (NSD), which approved of the study (ref. no. 22920/2).

## Results

The themes representing participants’ personal expectations, goals and hopes at the start of treatment at the CMHC are shown in Fig. 2 and they are reported in Biringer et al. [22]. At the start of treatment participants hoped to develop understanding of their mental health problems, find tools for coping (including medication), and they hoped to receive counselling and practical assistance from the health professionals with regard to family-, financial- and practical problems, the solution of which being deemed necessary to gain a safe basis for recovery in the long run. Most of the participants dreamed of ordinary independent lives, not needing help from others. A young woman, who struggled with severe anxiety and depressive thoughts, spending most of her days at home smoking cannabis, exclaimed:

*I want to manage by myself. I do not want special treatment!* (P2)

She and several others hoped to become able to carry out the usual everyday activities that belong to a normal life.

*I hope that I will be able to get up in the morning, and want to get up, want to eat, and want to do the worst things that I have to do…* (P2)

They hoped to become able to function around others, have a family and a job.

*My goal, it is… because there is so much… I want to be able to work, I want to be able to go out amongst people, I want to be able to talk to my child, I want to be able to take my child with me somewhere where there are people, without being a nervous wreck…* (P2)

Several participants, like this woman who had recently experienced an episode of psychosis and a longer stay at the ward, separated from her child, family and school, expressed hope for recovery.

*There is, getting a steady job and a family. I have my son and my boyfriend, so, to be able to move in together and not get sick again.* (P8)

*Isn’t that a good goal?* (EB)

*Yes, never give up hope!* (BS)

*No, there is hope even if you are sick, because you make the best of the situation. You think that you really don’t want to be admitted. But when you first are admitted, you just manage, at least that’s my experience [].* (P8)

*Can you tell us about what hope means to somebody who is really unwell and admitted? (EB)*

*It has a lot to say. Because if you give up, then, yes, maybe you don’t come out of the situation.* (P8)

Two years after the start of treatment, several participants offered positive stories about how they had achieved their recovery goals. For instance, a young man who at the first interview appeared as severely depressed and a bit delusional shared these goals and hopes with the interviewer during the first interview:

*In what way do you want the CMHC to help you today?* (EB)

*Erm… get me out of depression… and also help with my social anxiety …[], a practical job [], … finish my education. I have six subjects still to complete. []* (P10)

*Have you any other aims, like both for treatment and life you would like to mention?* (EB)

*Erm… Yeah. Get myself a girlfriend!* (P10)

Two years later he had achieved almost all of his goals from two years before, including getting a job, which he saw as his most important goal, and moving out of his parents’ home. He now appeared as a positive and eager person who willingly shared with the interviewers his personal journey from being a patient at a locked ward to having a good life with friends, his own place to live, and enjoying a full-time practical job. This was his spontaneous reaction as he was reminded of his aims from 2 years earlier:

*Yeah. If I remember correctly, then it’s just a girlfriend I am missing… (laughs)!* (P10)

In his experience the starting point for his recovery was the moment when a nurse at the ward he stayed at helped him contact the Labour and Welfare Administration. Later, as he continued seeing a therapist at the outpatient clinic, he experienced the attitude of his therapist as very supportive of his recovery. He experienced that the therapist was responsive to his everyday practical problems and interests. He and his therapist discussed practical aspects of work and leisure during therapy sessions. He used to use physical training as a means to help himself, and in particular he appreciated the therapist sharing their common interest in sports activities with him.

### Participants’ experiences of helpful help from health professionals at the CMHC

Six out of the eight participants felt that health professionals played a role in their recovery process. The participants experienced this role as helpful in a variety of ways. Several, like this young woman who struggled with depressive feelings, mentioned that just knowing that one had an opportunity to call the CMHC ‘if something happened’ or if one ‘needed to contact them’, was reassuring in and of itself.

*Did it help to be able to ring if things got difficult, or to know you could?* (EB)

*Yeah, [to know that] if there was a problem I can just go there, like…* (P3)

Another man with who struggled with alcohol problems and who used to go on 2–3 days drinking binges whenever his anxiety became to heavy to bear, also found the regular contact and talks with people from the CMHC useful. At the start of treatment he showed a ‘laidback’ attitude, his only goals being to manage to keep his scheduled appointments at the CMHC, stay sober during the planned diagnostic evaluations, and generally drink a bit less. During the next 2 years he got regular visits by the outreach team of the CMHC.

*Could you please say a little bit about how things are now?* (EB)

*Yeah, much better than before, to be honest. It has been OK to have somebody from the CMHC to speak to once a week or once a fortnight. They have helped me to get into rehab also, yeah, pretty much a positive thing that, yeah, actually works well. It’s alright to have somebody to speak to when you have bad days, or weeks, or months… Especially when the medication has no real effect either, then its really OK to have somebody to talk to. So that has managed to keep me going, then, so I have not felt too low. So, yeah, I cannot do anything other than give them [the outreach team] five stars.* (P 150)

Five themes were identified reflecting the participants’ experience of how health professionals at the CMCH met their expectations and how the help they received there was useful in their work towards their personal goals, as follows: A. Developing an understanding of oneself and one’s mental health problems; B. Learning how to change feelings and behaviours; C. Being ‘pushed’ into social arenas; D. Finding helpful medication; and E. Counselling in family, practical, and financial issues (Fig. 2). Additional file 1 summarises emerging themes and codes representing participants’ personal expectations, goals and hopes at start of treatment, experience of treatment and support of their goals from health professionals at the CMHC, and goals achieved two years after start of treatment.

#### A. Developing an understanding of oneself and one’s mental health problems

At the start of treatment, participants hoped that the health professionals would help them to understand why things had become difficult; why they had the thoughts and problems that they did; why they reacted like they did; and what they could do to avoid ending up like this again in future.

*That’s something that is good also, to learn more about myself and why those thoughts I’ve had, why, what could be the reason why I reacted the way I did.[] I want to learn more about myself, and why I ended up like I did and how I can avoid ending up like this again.* (P5)

Two years later, several were grateful for the explanations that their therapist had given for why things were difficult during their talks. For instance, the man above felt that the diagnosis his therapist had given him represented a useful explanation for his problems:

*Is it important to now have a diagnosis?* (BS)

*Not so much for the diagnosis in itself, in a way; however, it explains a number of things, that is, you understand why what has happened has happened.* (P5)

Several participants experienced that the professional helpers helped them see themselves and their problems more clearly and from new perspectives in the course of the therapy sessions. Some explained how their professional helpers had helped them become aware of their thoughts and feelings in certain situations and to analyse their own reactions related to the situations they encountered. One woman had these goals at the first interview:

*My aim is to be fine… better anyway …* (P1)

*Emm… For the pain?* (EB)

*Pain…- no, not just the pain – everything- all of me, you know? Because it is clear that how things are now, I’m irritable, I can be pissy and have short temper, I cry until I am despondent, and then… And of course it’s hard… So I wanna be well in all these ways, as well as I want to be well in my body…* (P1)

She described how she spent her energy on bitterness and ruminations about the past.

*I lost a boy many years ago, - he had leukemia and he died when he was two and a half, so I for sure have some of that still with me eating away… I should say, I will never be finished with that… Because you have one too few…so there’s like… bitterness and such from that time which is always there. Maybe it would be good for me to talk about all that stuff from the past… that it lies heavy and makes my life poorer, -right? You have a childhood that simply was not nice, and you got this with the kid….* (P1)

During her later regular talks with her therapist she experienced that her therapist explained the possible connection between grief and ruminations resulting from the earlier loss of her child and her current bodily pains.

*However, [the therapist] said: ‘But it is not certain that you’ve had time to think, because you had the kids you had at home and took care of them, and … and other stuff … So when they [the children] moved out, then you get more time to start thinking again and … take more things into you … ’.* (P1)

She described moments of sudden insights during the talks with her therapist.

*It’s obvious that the act of talking about things, and that the things are unmanageable… You are 100*% *safe [in the therapist’s office], right? And you put into words things that have bothered you… It is probably necessary. -Get it out… []*

*Did you feel that you got to talk about the important things with your therapist?* (EB)

*Yes, it was very important. And I had not really thought about it before myself either, before we got into it, you could say… then it was like: ‘Oh! God! Yeah […] I think there is … [something]’. She [the therapist] went into different stuff that I had not thought about…* (P1)

The talks in which she and her therapist went through the past made her feel very tired and worn, but they also revealed to herself that she needed to change herself.

*It is very tiring… And you get a little depressed by that, basically… You bring up things that you struggle with, right? And then it bothers you afterwards… Also, it is clear that, as I said to her [the therapist] after we had talked a couple of hours, that: ‘I just have to start thinking more about myself…’- Because I go around chronically feeling bad for everybody … And I feel like I’m not good enough in the eyes of others. And just that I can put it into words now that: ‘I must do something with myself…’* (P1)

In her experience the therapist also challenged her current patterns of thoughts and emotions with regard to her difficulties with putting her foot down and saying ‘No’ when family members demanded too much of her.

*There were situations when sort of, she, the psychologist, sort of asked when this and that occurred, yes, how I felt then. I did say one time, I remember that I felt totally like a whore and she understood this in fact, because the fact that you feel used all the time, right, and you cannot manage to say clearly that ‘enough is enough’. She illustrated this to me and showed me where I was, and that I was going backwards instead of forwards. So she made me aware of that. However, I have problems in carrying things through. I have got better at that, yes, indeed.* (P1)

Another participant spoke about how accepting things as they were helped her cope with things that had happened in the past.

*I have accepted some things, such that I have, for example, that my mother is no longer here, it is actually ok to a certain degree. […] Then I don’t have to constantly go round thinking about what could have been.* (P2)

In addition to experiencing the new insights about the link between body and soul, and their patterns of emotional reactions and behaviour in interpersonal relationships, participants learned how outer pressures, stress, or sleep problems could precipitate depressive reactions or psychotic experiences. Further, through discussions with professional helpers, some participants became more aware of early warning signs and triggers, and together with their therapist, several had planned what to do in case their problems worsened:

*We drew up a kind of plan during one of the recent appointments, or in any case, sort of, things I could do if I found myself in such a situation again […]. I have looked at this sheet since, so it is a good thing.* (P5)

Several participants described what they had learned from the professional helpers at the CMHC as the starting point for changing their emotions, thoughts and behaviour.

#### B. Learning how to change feelings and behaviours

At the start of treatment, some participants, like this man, said they hoped to learn ‘methods’ to better handle their negative emotions.

*To learn to work through things on my own, so that I don’t have to come back later. I feel that I can manage this myself, and I have lots of friends around me who support me, and my family supports me…Maybe it is just to open up now, so that it won’t ‘boil over.’* (P7)

Others wanted to learn how they could feel better and have more energy, cope with symptoms and distress, and deal with distressing thoughts and ruminations. During the course of treatment, several participants felt they learned ‘tools’ and ‘tricks’ from the professional helpers to avoid negative ruminations. One man described how their therapist had taught him to put negative thoughts aside and take one thing at a time.

*It is, in a way, to try to avoid the mill that just goes round and round and round, and in that way get out of a vicious circle. […] It is kind of to try to shut it in a cupboard and turn the key. Try to put things away and then try to do one thing at a time.* (P5)

The woman above with bodily pain who felt that ‘I must do something with myself’ explained that she learned to put her foot down when necessary during her sessions with her therapist and during the group course she attended.

*And I go with that endless bad conscience. And it was something that I practiced quite a bit on the course up there [at the CMHC], then. I have learned a bit from it. [] I have become much better at saying ‘No – I am busy’.[] And then it seeps in. But it has been damn tiring.*

*Seting boundaries?* (BS)

*Yeah, really clear boundaries.* (P1)

In the interview, she gave the example of her improved ability of refusing the demands her family put on her by describing how she said ‘No’ to her family when she went to her physical training group twice a week, leaving her phone at home so that they could not reach her.

Another woman, who came to the CMHC seeking help with handling her depressive thoughts and obsessive practices, felt that the relaxation techniques she learned from her helper at the CMHC were useful, as they made her calmer:

*I was really happy with her [the therapist], like the relaxation exercises, like meditation, she did so many different things… [These things] are very calming. It gave me, like, a clear head, not so much mess.* (P3)

*[] How long does it last when you have seen her, how long does the effect last?* (EB)

*Em, I guess, yeah, a few days I would say, maybe a week.* (P3)

The same also woman experienced that the medication she was prescribed helped her feel calmer.

#### C. Finding helpful medication

During the time since they started treatment several, but not all, of the participants found that the medication the professional helpers had prescribed helped them to feel calmer, have more positive thoughts, sleep better, or experience less racing thoughts.

*The last time you were here with us, you said that your expectations from the treatment at the centre were that your depression would be better, that you would get help with your obsessiveness and that the medication would work, what do you think about the goals now today?* (EB)

*Yeah, with the depression, the medication has really helped actually I think. [] They make me feel calmer. I get kind of more positive thoughts.* (P3)

A couple of participants who had used antidepressive medication experienced that these were part of the reason they managed to start engaging in normal activities, like physical training and socialising.

Some participants described ambivalence, however, about taking medication. Although they hoped the health professionals would help them to find medication that would reduce their emotional suffering, getting advice about how to stop taking the medication ‘when the time comes’ was just as important to these participants.

*I am on medication now, so I thought it might help me and, like, when I have to cut down and things like that, I think it’s a good idea to come here to the CMHC.* (P3)

Some felt disappointed and resigned at follow-up, as they perceived that they still needed to use medication to keep their mental health issues under control.

#### D. Being ‘pushed’ into social arenas

Several of the participants who experienced extreme discomfort in social situations hoped that their professional helpers would push them into such contexts to minimise their anxiety. They wanted the staff at the CMHC to ‘push’ them into social settings, such as shops and cafes:

*The only thing I can think of is to have training in being around people…being with people, and functioning in the context of a job and in the context of a school around people…* (P2)

At the second interview, several participants perceived that the helpers actually helped them to cope better with social anxiety by facilitating social activities outside of the CMHC. A few had been regularly accompanied to public places, like crowded streets or shops, by CMHC employees. In their experience, being among others became less unpleasant after some time was spent addressing such social challenges, and they no longer felt like ‘nervous wrecks’ at the thought of going out in public.

*What is it the people at the CMHC have done that has been helpful to you? (EB)*

*They have helped me with my social anxiety. That has come back now a little, when it comes to large crowds. So she [the mental health nurse] was with me a while. We went to the grocery store together. [] It was a bit forced on me, I felt, but it should feel that way, I guess. I do not know. It was not comfortable.* (P2)

Later in her process of recovery, partly due to the help from the mental health nurses from the CMHC who accompanied her among others, she became able to attend the rehearsals of the local brass band regularly. Playing an instrument among others in this band came to mean much to her.

*It is not just a band, it is often, for me at least that had some problems, it really helped to have some structured forms outside of school in a way. It is not so structured, yet you need to keep you reined in. And there are other people there, it’s not just you. That you are part of a whole means something…* (P2)

A young man was grateful for the scheduled weekly activity plan and the ‘Thursday-trips’ at the ward.

*Did it affect you physically or mentally, to get out in that way?* (EB)

*It challenged me […], but at the same time I felt safe…* (P10)

The man who used to go on a drinking binge whenever his anxiety became unbearable had previously not been able to leave his home when things were bad. In the past he had developed a strong dislike of therapist offices. In his experience, having CMHC employees visit him every one or two weeks was a good experience, as this meant he mixed with other people and talking with members of the outreach team helped to relieve his depressive feelings.

*It helps very well with a little chat. I am not really an office person. They exhaust me. I guess I have had too many hours in them, both to the doctor and … and principal and psychologists…* (P6)

*So that they come to your home, you think that’s good?* (EB)

*Yeah, its really good.* (P6)

*But what do you talk about when they come?* (EB)

*No, it’s anything and everything. From I have had a shitty week to them trying to help me a little bit of thinking ahead and, yes … discuss possible hospitalisation for a month or fortnight, just to get away a bit again. Because it helped the last time. After I was inside the rehab, so I have not been anywhere close to the quantity I used to drink. So it helped a lot.* (P6)

After two years of regular contact with the team members, he had come so far in his recovery that he was able to participate in volunteer work at the local Church Charity Centre, and he drank less than he used to two years earlier.

#### E. Counselling in family, practical and financial issues

Some participants said they wanted to ’function as other people do’ in terms of family life, education, and work; however, practical and financial challenges brought additional pressure to bear on their mental health. The interviews revealed that the participants’ expectations for support in terms of practical or financial issues related to becoming independent were not sufficiently met by the CMHC staff. For example, one young woman expressed at the first interview that she wished to complete her studies and start working, but at follow-up this had not yet come to fruition, as she had not completed her studies, did not have a job, and was living in a financial mess:

*Collecting the post is hell itself: […] There are twenty debt collection letters in there every time.* (P2)

One young man introduced earlier said he had talked so much about his desire for a job that the staff at the CMHC finally helped him contact the Labour and Welfare Administration.

*I’ve talked about the Labour and Welfare Administration all the time. I have said that: ‘I intend to work, I …’. - I’ve said that all along… And then I sort of talked so much about it, that they have helped me with that, then …* (P10)

To him, the staff helping him contact the Labour and Welfare Administration was the start of a long journey from being a patient at a locked ward to a full time employment he enjoyed two years later.

Several participants were facing severe family troubles, and it seemed like these problems either were causally related to or aggravated the emotional distress they also experienced. However, only two participants related that family members had been invited to meetings at the CMHC. In these instances, family consultations turned out to be positive, as they helped clarify or sort out interpersonal roles and problems.

One woman whose psychotic experiences had interrupted her education and family life said she wanted the talks at the CMHC to center more on present and future issues rather than problems in the past.

*I’ve talked very much [] about everything, past and stuff like that. So I really just want to move on, yes. Be more, how should I say, that it is about the present and the future rather than the past. (P8)*

*I get what you mean. But like now, have you discussed the future also?* (EB)

*Yes or the present. [] How I will be going forward, perhaps where I will be, whether I am going to go to school, and that I’m going to take a break now this spring and start again in the autumn, perhaps, when I’m ready for it*. (P8)

Two years later she spoke about how it helped as she and her therapist or her boyfriend together daydreamed about her future.

*It helps to dream a bit.* (P8)

*Do you do that with your therapist?* (EB)

*Yeah, I think about what i want to achieve.* (P8)

*You have it in your head, but do you share it with her or with anybody else?* (EB)

*Yeah, with my therapist, my boyfriend and…* (P8)

She was happy she started seeing a mental health nurse at the CMHC after having seen a psychologist for a while, as the nurse showed more interest in dealing with the practical aspects of her everyday life and her plans for her future.

### Expectations that were not met at the CMHC

Two participants reported that the measures carried out at the CMHC did not help with their mental health issues. For instance, one woman explained that her contact at the CMHC focused on issues other than those that were important to her; as a result, she had stopped confiding in her CMHC contact:

*There was probably something that she could help me with, but I stopped mentioning it after I had talked about it for a while… Sometimes when I said I needed help with something, we worked with something else. And that just left me thinking, ‘but I am not struggling with that’. […] I was not satisfied. Because I wanted help to sort things out.* (P2)

*And you did not get that?* (BS)

*No, it’s still all a mess, but they have tried though. I still have that feeling that I am a ‘lost cause’.* (P2)

Although seeming hopeful about her future at the first interview, she appeared disillusioned and frustrated at the second one.

*Stability, that’s all I want. A steady income, a house… An ordinary life is the biggest dream I have and I’m never gonna have that. When every tiny little thing is so hard, then it’s not that simple…* (P2)

One man, whose expectations at the start of treatment were to learn more about himself and early warning signals of relapse, methods of calming oneself or keeping things under control and getting off medication, had the following reaction after the interviewer at the second interview repeated his aims for treatment from two years prior:

*Do you feel like you’ve accomplished any of these?*

*Not at the CMHC.* (P4)

*Not there?* (EB)

*No, the only thing is maybe … that you can change the way you think. … Apart from that, well, none of the goals were achieved at the CMHC.* (P4)

*Not in the CMHC, but eitherway you have achieved it, you have learned more about yourself and signals?* (BS)

*Yeah, its like you gradually do a little more and a little more. [] I have learned a bit more about myself, but not… I think I would have done that without help also though.* (P4)

*Because in a way you learn that in life and not with a therapist you mean?* (EB)

*Yeah, I can learn with a therapist also I think, but I haven’t felt that so much recently though.* (P4)

In the interviews it appeared as if participants’ inner development and change in feelings and behaviours happened as a result of input from their health professionals and personal experiences outside of the context of the CMHC, and that the process of change, or growth, was very much influenced by life events and contextual factors.

### Life events, meaningful activities and contextual factors

In addition to explaining how they experienced the helpfulness of their contacts at the CMHC in their work towards their recovery goals, participants also reported that contextual factors (e.g. school or supported work programmes), meaningful activities (such as playing music or repairing cars) or life events (e.g. having a baby or finding a partner) had added new meaning to their lives and contributed to their inner personal processes of growth and maturity. One young woman felt that becoming a mother had made the biggest difference in her life.

*But about when you feel bad due to stress and your head is in chaos, do you see some difference in how you tackle this now compared with two years ago?* (EB)

*Yes, there is yeah.... when you have somebody to live for…* (P2)

Her son added new meaning to her life and helped her challenge her anxiety in social situations, as she brought him with her when she had to go to shops and public places.

## Discussion

The study provided an in-depth exploration of how service users with mental health problems experience the help they are offered from therapists or other health professionals at a typical CMHC. Participants’ experiences of how professional helpers had supported them in their struggle towards their personal recovery goals varied. However, our findings confirm that recovery is a subjective and comprehensive process driven by the person’s own goals and desires. The help from the CMHC that the participants found most useful were as follows: help with understanding themselves and their emotional problems, reactions and ‘warning signals’; being advised on how to change emotional reactions and behaviours; and feeling supported and motivated in challenging the restrictions that anxiety placed on them in social situations in their everyday lives. These approaches are central components of clinical approaches frequently used by health professionals in the type of CMHC where this study was set. Service users shared similar experiences in earlier studies showing that explanations for and insight into mental health problems [32–34], learning coping mechanisms, changing thinking patterns, and dealing with stress and early signals of worsening are helpful [34–38]. Most of these comparable studies explored service users’ experiences between or after acute phases of illness, and the ways participants were recruited and the types of services used varied. In the present study participants were interviewed two years after they first started treatment in specialist care. Although most participants in the study were young (five out of eight were younger than 30 years of age) and they were recruited at the specialist centre relatively early after the start of their mental health problems, compared to participants in most of the studies in the field [34, 35, 39], it may be that the valued approaches reported by the participants in the present study are more useful during later stages of recovery than during acute stages of illness.

### Personal goals and hopes as driving forces in recovery

Health professionals’ ability to inspire and maintain hope is viewed as playing a central role in the motivational resources necessary for recovery [40–42]. Having life goals increases service users’ motivation for treatment [43], and such goals contribute to health and well-being [43, 44]. A recent study by Sælør et al. [45] showed how setting goals and reaching them inspired hope in participants struggling with mental health issues. Trust in relationships with others, and receiving help with practical challenges and bringing order to economic chaos, inspired hope for the future. The present study also emphasised personal aims and hopes as a core element in recovery [2–4]. Most participants showed a goal-oriented attitude. Expectations and hopes related to symptom relief and life goals were overlapping and served as driving forces in the participants’ personal struggles towards a better life. The perceived usefulness of the help and support the participants had received at the CMHC was judged as much according to whether it had proven to be helpful in the participants’ own work towards life goals (such as completing education or having a job) or better role functioning (i.e., functioning in the family or in social contexts) as according to whether it had provided relief from symptoms or suffering. This finding supports previous research showing that consumer satisfaction correlates more with improvements in self-identified problems than improvements in symptom and function in the secondary mental health care service [46]. Earlier recovery studies have also suggested that for a person who suffers from mental health issues, social relations, productive activities, and resuming control over his or her life may be just as important goals as amelioration of symptoms [8, 14, 16, 18, 22]. Indeed, many mental illness–related problems dissipate when everyday problems are resolved [8].

### Personal recovery as a comprehensive and contextual process

Personal recovery is a unique process that does not necessarily take place in the therapist’s office; rather, it can also occur within the context of the person’s everyday life [2, 47–49]. In other words, a person’s treatment and recovery cannot be viewed as distinct from his or her life and ambitions [8, 33, 48]. Borg and Kristiansen [47] earlier found that dedicated helpers available to address challenges in various aspects of everyday life were of great help for persons with severe mental issues. Participants in the study by Sælær et al. [45] experienced that receiving help with practical challenges and bringing order to economic chaos inspired hope for the future. However, an important finding in the present study, was that the treatment participants received had not been helpful for important issues they struggled with. As in the study by Morgan et al. [12], some reported ‘miscommunication’ in terms of feeling that the therapist did not work on the things most important to the individual. Although coping, empowerment, and a comprehensive approach to the service users’ needs are supposed to be key elements of the Norwegian CMHCs [50], many of the participants felt that their expectations were not sufficiently met in terms of receiving counselling and practical assistance for interpersonal problems and practical issues related to becoming independent (e.g. completing education, starting work, solving interpersonal and financial problems, and having a home and family). Individuals’ social and material resources are an important platform for recovery [51–53], and for the participants in the present study, sorting out these issues may contribute to a safe and healthy basis for recovery in the long run. From a healthcare system perspective, recovery-supporting interventions related to education, work, and financial issues may be the responsibility of community services or the Labour and Welfare Administration; however, specialist services should be aware of their responsibility for incorporating individuals’ goals into treatment, including those linked to education, work, family life, social roles, and material issues, as well as providing information about and coordinating the specific interventions deemed necessary by the individual service user to achieve his or her aims within these domains.

### Strengths and limitations of the study

The study was limited in that it included a small number of participants; the transferability of findings may therefore be called into question. However, the typical setting of a CMHC and the common types of mental health issues explored support the transferability of the results. Further, the process of engaging in an on-going reflexive analysis is difficult [54]. Two of the researchers who planned and performed the study had professional backgrounds in mental health care, and although they were aware of how their preconceptions and attitudes could affect the questions asked and conclusions drawn, intersubjective elements may have influenced data collection and analysis. However, active participation by the co-researcher with experience as a service user during the interviews and analysis helped the researchers with professional backgrounds to better understand what participants were trying to communicate. To ensure the internal validity of the findings and conclusions, the raw data were continually revisited to confirm that any interpretations were grounded in the participants’ interviews. Finally, the information was partly retrospective, and we cannot exclude the possibility of recall bias in the information provided.

## Conclusions

The service users felt that their expectations about treatment had been met in that they had been helped to better understand themselves and their problems, change their feelings and behaviours, challenge their social anxiety and find helpful medication. Unfortunately, their expectations about counselling and practical assistance with regard to family, practical, and financial issues that needed to be solved to achieve a better life in the long run were insufficiently met at the CMHC. In the light of current policy guidelines focusing on comprehensive person-centred approaches, CMHCs should reconsider their role as facilitators of service users’ personal expectations for recovery. To support recovery, health professionals should acknowledge the individual’s hopes and aims and take a more comprehensive approach to his needs and desires. This, in particular, means facilitating practical help and counselling with regard to education, work, and financial problems and includes service users’ families and social networks, which are all factors that constitute a basis for recovery in the long run.

## Additional file

Additional file 1: Participants’ expectations, goals and hopes at start of treatment, experience of treatment and support of their goals from health professionals at the CMHC, and goals achieved two years after the start of treatment: Overview of themes and codes. (DOCX 30 kb)

## Abbreviations

CMHC: Community Mental Health Centre
