# Supplementary material for: Experiences of support in working toward personal recovery goals: a collaborative, qualitative study
Source: BMC Psychiatry. 2016 Nov 25;16:426. doi: 10.1186/s12888-016-1133-x (PMC5124226; doi:10.1186/s12888-016-1133-x)
Supplement: Additional file 1: — Participants’ expectations, goals and hopes at start of treatment, experience of treatment and support of their goals from health professionals at the CMHC, and goals achieved two years after the start of treatment: Overview of themes and codes. (DOCX 30 kb) [file 12888_2016_1133_MOESM1_ESM.docx]

**Additional file 1** Participants’ expectations, goals and hopes at start of treatment, experience of treatment and support of their goals from health professionals at the CMHC, and goals achieved two years after the start of treatment: Themes and codes

| ***P*** | **Expectations, goals and hopes at the start of treatment** | **How the CMHC met expectations and helped to achieve goals and hopes** | **Expectations met, goals and hopes achieved at follow-up** |
| --- | --- | --- | --- |
|  |  | ***«Helping me see things that I don’t see»*** |  |
|  |  | **A. Developing an understanding of oneself and one’s mental health problems** |  |
| **1** | Expected to talk about the problems to get rid of them (but understands that she must also help herself)  Wanted to learn to see things that she does not see clearly herself, but feels she needs someone to show her the way so that she can become able to help herself  Wished to clarify whether she actually suffered from a mental health problem that may explain her constant bodily pains | The therapist helped her to see herself differently  Received help with thinking things through as opposed to merely accepting them  Learned to become aware of thoughts and feelings in certain situations, as well as how to analyse episodes and reactions  Developed the insight that mental and physical problems go hand in hand | Analyses situations the way she learned from her therapist to avoid negative feelings and bad conscience  Earlier, she saw things in ‘black-and-white’, now she sees nuances and sees more through situations  Has not found an explanation for her chronic pain |
| **4** | Expected help with getting to know himself better because knowing oneself is just as important as medication  Hoped to be given tools to keep problems away; in particular, he wanted to learn mindfulness techniques to control problems and mood swings  Expected to learn about signals suggesting that things are not as they should be | Does not feel that he learned much at the CMHC (maybe with the exception that one can change the way one thinks)  Learnt a lot more from a book about mindfulness than from the health professionals | Has learned more about himself and his own warning signals, but does not feel this knowledge came from the CMHC – rather, it came from himself (as this is something one learns in life) and from material he found on the internet and in books |
|  |  | ***«I must do something about myself»*** |  |
|  |  | **B. Learning how to change feelings and behaviours** |  |
| **5** | Hoped to get help with sorting out his thoughts  Waned to learn what he can do to prevent himself from ending up like this again  Hoped to learn how to improve his energy levels | From his therapist, he learned tricks to avoid negative ruminations and put negative thoughts aside  Learned to take one thing at the time so that he will not again become overwhelmed with the problems and ruminations that brought on his depression  Together with his therapist, he made a plan for what to do to avoid a relapse of mental distress | Has become better at sorting out his own problems and has a written plan for what to do in case he finds himself in such a situation again  Manages his work and his relationship with his wife  Does not have more energy  Has accepted that he has to live with the negative chaotic thoughts that overwhelm him in stressful situations |
| **8** | Wished to learn how to cope with the stressful feelings she experiences on a daily basis | Her therapist taught her how to take one thing at the time  She learned to become aware of symptoms and to slow down if she felt distressed  At the course, she learned breathing exercises and relaxation techniques  Staff at the ward encouraged her to go for walks, which she felt helped kept her going | Is more aware of symptom triggers  Manages to lead a regular life, taking one thing at the time instead of doing a thousand things at once  Whenever she feels distressed, she focuses on sleep and regular meals, tries to do things she likes and contacts her doctor to increase her medication dose |
|  |  | ***«Medication really helped»*** |  |
|  |  | **C. Finding helpful medication** |  |
| **3** | Hoped to receive medication that helps  When the time is right, she wants help with reducing medication | Medication helped her to feel more calm, have more positive thoughts | Feels well and manages her daily life  Is disappointed that she has not yet been able to discontinue her medication |
|  |  | ***«Challenging myself»*** |  |
|  |  | **D. Being ‘pushed’ into social arenas** |  |
| **10** | Expected that the staff will help him with his social anxiety by pushing him to go to stores and cafes | He enjoyed the regular activity plan and trips at the ward which helped him to challenge the discomfort he experienced when he had to go out in public  He felt more safe when he went with people sharing the same problems | Does not have feelings of discomfort in social situations  Has several friends  Manages a regular fulltime job in which he has regular contact with others |
|  |  | ***«Stable income, a house, -an ordinary life is the biggest dream I have»*** |  |
|  |  | **E. Counselling in family, practical and financial issues** |  |
| **8** | Wanted the therapist to focus on present and future issues, like where she should be and what she should do  Desired advice and help with planning how to complete her education and whether she should have another child  Her aims were to complete her studies and get a job; avoiding disability pension was an aim in itself  Hoped that she will be able to move in with her boyfriend | Discussing practical everyday issues was useful  She and her therapist daydreamed about her future life (because dreaming gave her hope)  Changing from a psychologist to a nurse was alright, as nurses are less analytical and more focused on practical issues | Has found work (part-time supported employment in the community) with the help of the Labour and Welfare Administration  Has avoided disability pension  Stays with her boyfriend for longer intervals  Has not had another child |
| **10** | Hoped to manage a practical job  Wanted to finish his educational degree  Aimed at moving out of his parents’ home and into his own place  Dreamed of finding a girlfriend  Wished to get on with his life | Staff members helped him to find information about an assisted training work course and contacting the local Labour and Welfare Administration office | Has a regular fulltime job  Has his own apartment  Has not yet achieved his goals of finding a girlfriend and completing his academic degree |
